# From Gigabyte to Kilobyte: A Bioinformatics Protocol for Mining Large RNA-Seq Transcriptomics Data

**DOI:** 10.1371/journal.pone.0125000

**Published:** 2015-04-22

**Authors:** Jilong Li, Jie Hou, Lin Sun, Jordan Maximillian Wilkins, Yuan Lu, Chad E. Niederhuth, Benjamin Ryan Merideth, Thomas P. Mawhinney, Valeri V. Mossine, C. Michael Greenlief, John C. Walker, William R. Folk, Mark Hannink, Dennis B. Lubahn, James A. Birchler, Jianlin Cheng

**Affiliations:** 1 Computer Science Department, University of Missouri, Columbia, Missouri, United States of America; 2 MU Botanical Center, University of Missouri, Columbia, Missouri, United States of America; 3 Division of Biological Sciences, University of Missouri, Columbia, Missouri, United States of America; 4 Department of Biochemistry, University of Missouri, Columbia, Missouri, United States of America; 5 Department of Chemistry, University of Missouri, Columbia, Missouri, United States of America; 6 Informatics Institute, University of Missouri, Columbia, Missouri, United States of America; 7 C. Bond Life Science Center, University of Missouri, Columbia, Missouri, United States of America; University of Toronto, CANADA

## Abstract

RNA-Seq techniques generate hundreds of millions of short RNA reads using next-generation sequencing (NGS). These RNA reads can be mapped to reference genomes to investigate changes of gene expression but improved procedures for mining large RNA-Seq datasets to extract valuable biological knowledge are needed. RNAMiner—a multi-level bioinformatics protocol and pipeline—has been developed for such datasets. It includes five steps: Mapping RNA-Seq reads to a reference genome, calculating gene expression values, identifying differentially expressed genes, predicting gene functions, and constructing gene regulatory networks. To demonstrate its utility, we applied RNAMiner to datasets generated from *Human*, *Mouse*, *Arabidopsis thaliana*, and *Drosophila melanogaster* cells, and successfully identified differentially expressed genes, clustered them into cohesive functional groups, and constructed novel gene regulatory networks. The RNAMiner web service is available at http://calla.rnet.missouri.edu/rnaminer/index.html.

## Introduction

Transcriptome analysis is essential for determining the relationship between the information encoded in a genome, its expression, and phenotypic variation [1,2]. Next-generation sequencing (NGS) of RNAs (RNA-Seq) has emerged as a powerful approach for transcriptome analysis [3,4] that has many advantages over microarray technologies [5,6,7].

A RNA-Seq experiment typically generates hundreds of millions of short reads that are mapped to reference genomes and counted as a measure of expression [5]. Mining the gigabytes or even terabytes of RNA-Seq raw data is an essential, but challenging step in the analysis.

In order to address these challenges, RNAMiner has been developed to convert gigabytes of raw RNA-Seq data into kilobytes of valuable biological knowledge through a five-step data mining and knowledge discovery process. RNAMiner integrates both public tools (e.g., TopHat2 [8], Bowtie2 [9], Cufflinks [10], HTSeq [11], edgeR [12], and DESeq2 [13]) with our in-house tools (MULTICOM-MAP [14,15,16]) to preprocess data and identify differentially expressed genes in the first three steps. In the last two steps, RNAMiner uses our in-house tools MULTICOM-PDCN [17,18] and MULTICOM-GNET [19,20] to predict both functions and gene regulatory networks of differentially expressed genes, respectively.

As proof of principle, we have applied the RNAMiner protocol to RNA-Seq data generated from *Human*, *Mouse*, *Arabidopsis thaliana*, and *Drosophila melanogaster* cells. The data mining process successfully produced valuable biological knowledge such as differentially expressed genes, cohesive functional gene groups, and novel hypothetical gene regulatory networks by reducing the size of the initial data set over a thousand-fold.

## Methods

Some RNA-Seq data analysis pipelines (e.g. Galaxy [21], KBase [22], iPlant [23]) provide users with a convenient and free platform for RNA-Seq data analysis by combing public tools, such as TopHat [24], Bowtie [25], Cufflinks [10], Cuffmerge [10], and Cuffdiff [10]. As with these pipelines, RNAMiner combines these public tools such as TopHat2 [8], Bowtie2 [9], Cufflinks [10], Cuffdiff [10], and it is free. However, there are several differences between RNAMiner and other pipelines. First, RNAMiner integrates more tools, such as HTSeq [11], edgeR [12], DESeq2 [13], and our in-house MULTICOM-MAP [14,15,16], to calculate gene expression values and identify differentially expressed genes. These tools can generate more accurate consensus results. For example, RNAMiner uses Cuffdiff, edgeR, and DESeq2 to identify differentially expressed genes based on TopHat mapping results and gene expression values calculated by HTSeq and MULTICOM-MAP. RNAMiner generates up to five distinct lists and one consensus list of differentially expressed genes, which usually produces more accurate results. Second, RNAMiner predicts functions of differentially expressed genes and builds gene regulatory networks by integrating our in-house tools MULTICOM-PDCN [17,18] and MULTICOM-GNET [19,20]. These analyses provide more biological information. Other pipelines (e.g. Galaxy and iPlant) do not provide these analyses. Another software package—KBase—contains a service to predict gene functions, but the service only provides GO annotation for plant genomes. Third, without requirements for user registration and selection of many parameters, RNAMiner is easier to use than other pipelines. Compared to running each tool separately, users can easily run all these tools integrated in RNAMiner at one time and download results generated by all the tools at the RNAMiner web site.

**Fig 1:**
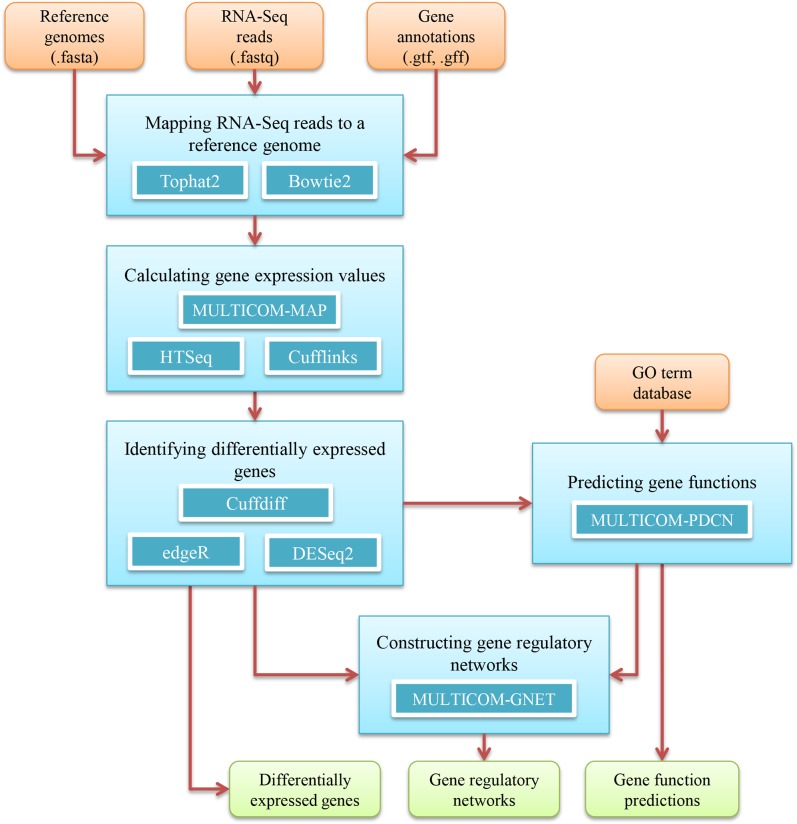
The RNAMiner protocol for big transcriptomics data analysis. Five blue boxes denote five data analysis steps, i.e. mapping RNA-Seq reads to a reference genome, calculating gene expression values, identifying differentially expressed genes, predicting gene functions, and constructing gene regulatory networks. The tools used in each step are listed inside each box. The external input information is represented by brown boxes and the final output information is represented by green boxes. The information flow between these components is denoted by arrows.

The five data analysis steps of the RNAMiner protocol (Fig 1) are described individually in sub-sections below. Tables 1 and 2 list the versions and the parameters of all the public tools used in RNAMiner and describe the meanings of the parameters.

**Table 1:** The versions of the public tools used in RNAMiner.

Tool	Version
TopHat2	2.0.6
Bowtie2	2.1.0
Cufflinks	2.2.1
HTSeq	0.5.3p7
edgeR	3.4.2
DESeq2	1.2.10

**Table 2:** The parameters of the public tools used in RNAMiner, the parameter values, and the descriptions.

Tool	Parameter	Value	Description
**TopHat2**	—read-mismatches	2	The maximum number of mismatched nucleotides between a read and a reference allowed for a valid mapping.
	—read-gap-length	2	The maximum number of gaps in the alignment between a read and a reference genome allowed for a valid mapping.
	—splice-mismatches	0	The maximum number of mismatches allowed in the "anchor" region of a spliced alignment.
	—segment-mismatches	2	Read segments are mapped independently, allowing up to this number of mismatches in each segment alignment.
	—segment-length	25	A read is cut into segments each having at least this length. These segments are mapped independently.
**Bowtie2**	—end-to-end		In this mode, Bowtie2 requires that the entire read to be aligned from one end to the other, without any trimming (or "soft clipping") of characters from either end. Local alignment is not used in Bowtie2.
	—sensitive		This option generally balances speed, sensitivity and accuracy.
**Cufflinks**	—frag-len-mean	200	The expected (mean) fragment length.
	—frag-len-std-dev	80	The standard deviation of fragment lengths.
	—min-isoform-fraction	0.10	Suppress isoform transcripts below this abundance level.
	—pre-mrna-fraction	0.15	Suppress intra-intronic transcripts below this level.
	—max-intron-length	300000	Ignore alignments with gaps longer than this.
**Cuffdiff**	—min-alignment-count	10	Minimum number of alignments in a locus for testing.
	—FDR	0.05	The maximum false discovery rate allowed after statistical correction.
	—frag-len-mean	200	The expected (mean) fragment length.
	—frag-len-std-dev	80	The standard deviation of fragment lengths.
**HTSeq**	-a	10	Skip all reads with alignment quality lower than this minimum value.
	-t	Exon	Feature type (3rd column in GFF file) to be used. All the features of other types are ignored.
	-i	gene_id	GFF attribute to be used as feature ID.
	-m	Union	Mode to handle reads overlapping more than one feature.
**DESeq2**	Test	LRT	Use the likelihood ratio test on the difference in deviation between a full and reduced model formula (nbinomLRT).
	fitType	parametric	The type of fitting of dispersions to the mean intensity. Parametric: fit a dispersion-mean relation via a robust gamma-family GLM.
**edgeR**	Pair	NULL	First two levels of object$samples$group (a factor) are used in comparison.
	Dispersion	NULL	Either the common or tagwise dispersion estimates from the DGEList object will be used, according to the value of common.disp.
	common.disp	TRUE	Testing carried out by common dispersion for each tag/gene.

### 1. Mapping RNA-Seq reads to a reference genome

**Fig 2:**
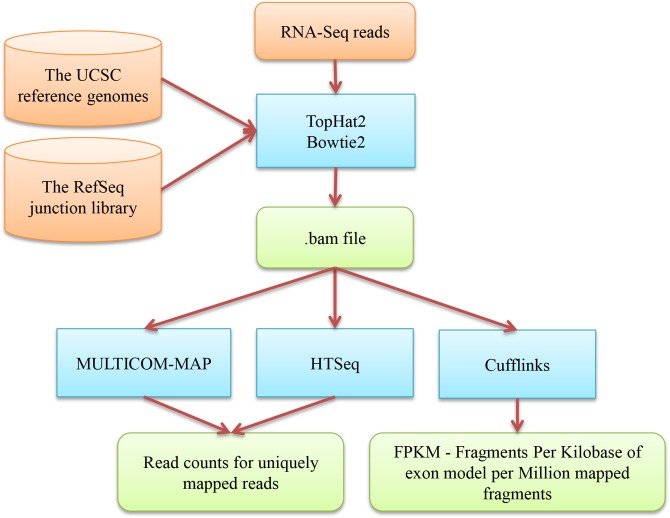
The workflow of mapping RNA-Seq reads to a reference genome and calculating gene expression values. The blue boxes denote the tools (TopHat2, Bowtie2, MULTICOM-MAP, HTSeq, Cufflinks) used in the steps of mapping RNA-Seq reads to a reference genome and calculating gene expression values. The external input information is represented by brown boxes and the output information is represented by green boxes. The information flow between these components is denoted by arrows.

We use two public tools, TopHat2 [8] and Bowtie2 [9], to map RNA-Seq reads to reference genomes in the UCSC genome browser [26] in conjunction with the RefSeq genome reference annotations [27]. The workflow of mapping RNA-Seq reads to a reference genome and calculating gene expression values is illustrated in Fig 2. It is worth noting that, since the RefSeq genome reference annotations contain information about some non-coding small RNAs, the reads of the non-coding RNAs are mapped and counted in addition to regular protein coding mRNAs. MULTICOM-MAP [14,15,16] is used to remove reads mapped to multiple locations in a reference genome from the mapping data in BAM/SAM format [28] generated by TopHat2 and Bowtie2. Only reads mapped to a unique location on the genome are retained to calculate the read counts of the genes. We use MULTICOM-MAP to analyze the mapping results to obtain baseline information, such as the total number of reads, the number of reads mapped to a unique location, and the number of reads mapped to multiple locations. This mapping process can generally reduce the size of datasets by *several* orders of magnitude.

### 2. Calculating gene expression values

For RNAMiner, MULTICOM-MAP [14,15,16] and two public tools: HTSeq [11] and Cufflinks [10] are used to calculate gene expression values according to the genome mapping output and the RefSeq genome reference annotation [27]. MULTICOM-MAP and HTSeq produce raw read counts, while Cufflinks generates normalized values in terms of FPKM, i.e., fragments per kilobase of exon model per million mapped fragments. The normalized gene expression values generated by Cufflinks are used to identify differentially expressed genes in the next step. The read counts generated by MULTICOM-MAP and HTSeq are fed separately into two R Bioconductor packages, edgeR [12] and DESeq2 [13], to identify differentially expressed genes. The normalized gene expression values (RPKM, reads per kilobase of exon model per million mapped reads) of MULTICOM-MAP are used to construct gene regulatory networks in the last step. Cufflinks, MULTICOM-MAP, and HTSeq are largely complementary and mostly differ in how they handle the reads mapped to common exons of multiple isoforms of a gene. Cufflinks distributes the count of such reads to each isoform proportionally according the estimated probability that the reads were derived from the isoform. In contrast, MULTICOM-MAP distributes the total count of such reads to each isoform, while HTSeq discards the reads without counting them for any isoform. This analysis step generates the overall expression profile of most genes in a transcriptome and can reduce the size of data from Step 1 by ~one thousand-fold, from gigabytes to several megabytes.

### 3. Identifying differentially expressed genes

**Fig 3:**
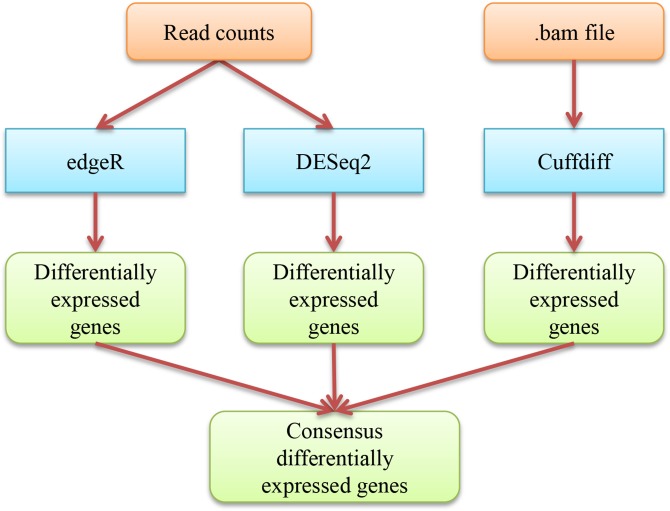
The workflow of identifying differentially expressed genes. The blue boxes denote the tools (edgeR, DESeq2, Cuffdiff) used in the step of identifying differentially expressed genes. The external input information is represented by brown boxes and the output information is represented by green boxes. The information flow between these components is denoted by arrows.

We use Cuffdiff [10] and two R Bioconductor packages, edgeR [12] and DESeq2 [13] to identify differentially expressed genes separately (see Fig 3 for the workflow). EdgeR and DESeq2 identify differentially expressed genes based on the raw read counts calculated by MULTICOM-MAP and HTSeq, resulting in four lists of differentially expressed genes (i.e., edgeR+MULTICOM-MAP, edgeR+HTSeq, DESeq2+MULTICOM-MAP, and DESeq2+HTSeq). In contrast Cuffdiff identifies differentially expressed genes directly from the genome mapping outputs containing only reads mapped to a unique location on the genome, resulting in one list of differentially expressed genes. Cuffdiff, edgeR and DESeq2 further adjust p-values by multiple testing using Benjamini and Hochberg's approach, which controls the false discovery rate (FDR) [10,12,13]. Usually, the cut-off of p-value (or q-value) is set to 0.05. Based on the five lists of differentially expressed genes generated by Cuffdiff, edgeR+MULTICOM-MAP, DESeq2+MULTICOM-MAP, edgeR+HTSeq, and DESeq2+HTSeq, a consensus list of differentially expressed genes is generated as the final output which usually comes from the overlap of at least three lists of differentially expressed genes. This step generates valuable information that may play an important role in the biological experiment. For example, the significantly differentially expressed genes identified by RNAMiner could be the targets for new biological experiments. This analysis step can generally reduce the size of data of the previous step by a couple orders of magnitude, condensing the data set size to several hundred kilobytes.

### 4. Predicting gene functions

**Fig 4:**
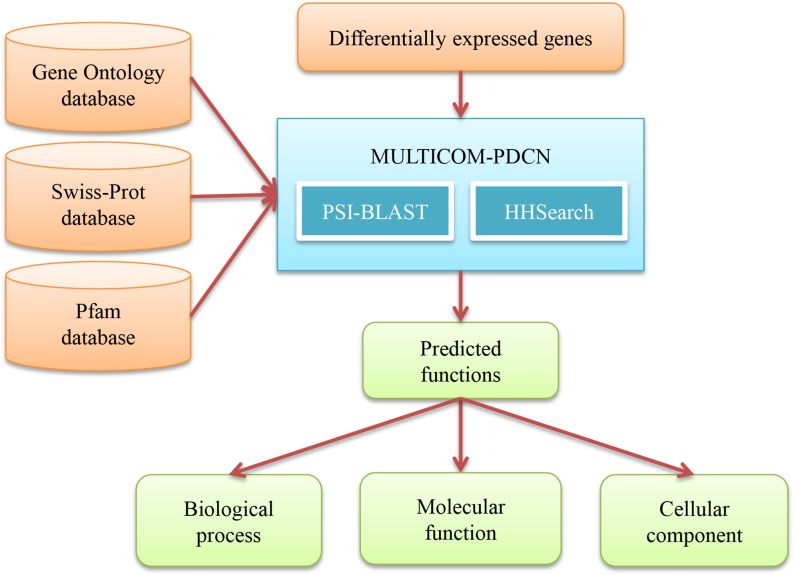
The workflow of predicting gene functions. The blue box denotes the tool used in the step of predicting gene functions. The tools of PSI-BLAST and HHSearch used in MULTICOM-PDCN are listed inside the blue box. The external input information is represented by brown boxes and the output information is represented by green boxes. The information flow between these components is denoted by arrows.

We use MULTICOM-PDCN [17,18], a protein function prediction method ranked among the top methods in the 2011–2012 Critical Assessment of Function Annotation (CAFA) [29], to predict functions of differentially expressed genes (see Fig 4 for the workflow). MULTICOM-PDCN integrates sequence-profile and profile-profile alignment methods (PSI-BLAST [30] and HHSearch [31]) with protein function databases such as the Gene Ontology database [32], the Swiss-Prot database [33], and the Pfam database [34], to predict functions of proteins in Gene Ontology [32] terms in three categories: biological process, molecular function, and cellular component. MULTICOM-PDCN also provides some statistical information about the predicted functions, such as the number of differentially expressed genes predicted in each function. We then use the Cochran-Mantel-Haenszel test implemented by R program mantelhaen.test [35,36] to check if predicted function terms are good for Fisher’s exact test to identify the significantly enriched GO function terms. A p-value from the MH test lower than 0.05 suggests the two nominal variables (e.g., two function terms) are conditionally independent in each stratum [35,36]. We then calculate a p-value of enrichment for each predicted function using R function fisher.test [35,36,37,38,39,40,41,42] and sort the predicted functions by their p-value in ascending order, from the most significant ones to the least significant ones. The list of the most significantly enriched functions can provide an overview of the biological processes differentially perturbed in two biological conditions. Although the physical size of the data and knowledge generated in this step is comparable to the size of the data in the previous step, the differentially expressed genes can be organized in three functional perspectives: biological process, molecular function, and cellular component.

### 5. Constructing gene regulatory networks

**Fig 5:**
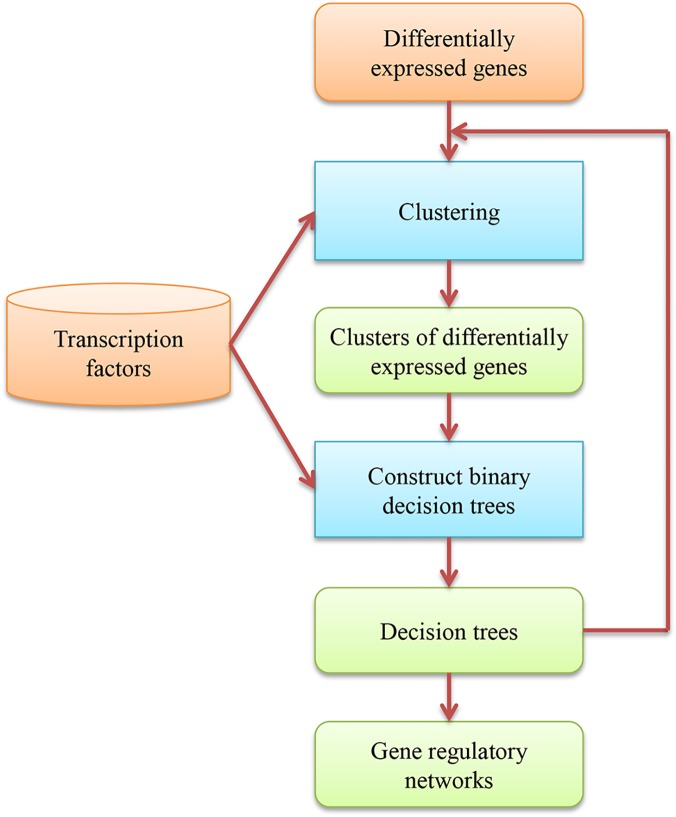
The workflow of constructing gene regulatory networks. The blue boxes denote the methods used by MULTICOM-GNET in constructing gene regulatory networks. The external input information is represented by brown boxes and the output information is represented by green boxes. The information flow between these components is denoted by arrows.

We use MULTICOM-GNET [19,20] to construct gene regulatory networks based on differentially expressed genes and transcription factors in a genome (see Fig 5 for the workflow). MULTICOM-GNET firstly clusters differentially expressed genes with similar expression patterns into functional clusters using the K-means clustering algorithm. Secondly, it builds a binary decision tree to represent potential regulatory relationships between several selected transcription factors (TFs) and the genes in each cluster. Thirdly, it re-assigns differentially expressed genes into clusters whose gene regulatory tree best explained the expression patterns of the genes. The last two steps are repeated until the maximized likelihood of the gene expression data is reached [19,20]. We also use a R network analysis and visualization package “igraph” [43] to visualize gene regulatory networks by linking the regulatory relationships between and within all the gene regulatory modules predicted by MULTICOM-GNET together. The regulatory network construction step generates a comprehensive understanding of underlying mechanisms controlling the expression of a transcriptome and can significantly reduce the size of data. The hundreds of kilobytes of the biological network data provide a system view of the cellular systems, which can be more readily utilized to generate valuable hypotheses for biological experiments.

For replicates from RNA-Seq experiments, RNAMiner maps reads of the replicates to reference genomes and calculates gene expression values separately. The gene expression values of the replicates of two samples are combined into a profile (i.e. a vector of the expression values of a gene in each replicate of each condition), which is input into edgeR and DESeq2 to identify differentially expressed genes. Additionally, the TopHat mapping results of the replicates of two samples are input into Cuffdiff to identify differentially expressed genes. EdgeR [12], DESeq2 [13], and Cuffdiff [10] handle the replicates by modeling the variance (dispersion) in counts across the replicates as a function of the mean count of the replicates. EdgeR [12] estimates the variance by conditional maximum likelihood conditioned on the total count for the gene. DESeq2 [13] uses a flexible and mean-dependent local regression to estimate the variance between the replicates by pooling genes with similar expression levels to enhance the variance estimation. Cuffdiff [10] estimates the variance based on a negative binomial model and uses t-test to calculate the test statistics. Cuffdiff can make a model on each condition with replicates, or use a global model for all conditions together.

Before calling a tool to do data analysis, RNAMiner checks whether the data is appropriate to the tool. For example, MULTICOM-GNET [19,20] is not applied if no transcription factors exist in differentially expressed genes because MULTICOM-GNET needs at least one transcription factor to build gene regulatory networks. Another example is, for some special datasets, overexpression of some treatments in some regions of the genome in one condition leads to very large read counts of some genes in this condition, and dramatic differences of gene expressions between two conditions. This violates the assumption of edgeR’s normalization method [12] that the majority of the genes should have similar expression levels. Therefore, calculating a normalization factor across all loci is difficult. RNAMiner will check this assumption and will not call edgeR if it is violated.

## Evaluation and Discussion

We tested the RNAMiner protocol on six sets of RNA-Seq data generated from *Human*, *Mouse*, *Arabidopsis thaliana* and *Drosophila melanogaster* cells in order to evaluate its effectiveness. The details such as organisms, biological conditions, and experimental settings about the six sets of RNA-Seq data were reported in Table 3. The results of each of the five analysis steps are described and discussed as follows.

**Table 3:** The organisms, conditions, replicate numbers, and descriptions of the six sets of RNA-Seq data.

Data set	Organism	Conditions	Replicates	Description
**First**	Mouse	Control, two botanicals (Lessertia frutescens and Sambucus nigra), and Nrf2 activator CDDO (2-cyano-3, 12-dioxooleana-1, 9-dien-28-oic acid)	No	Two mouse fibroblast cell lines: mutant (PGAM5 knock-out) mouse and wild-type mouse.
**Second**	Mouse	FruHis (0, 1, 2, 4, 8, 16 mM) in the absence (samples 2A, 2B, 2C, 2D, 2E, 2F) or presence (samples 2G, 2H, 3A, 3B, 3C, 3D) of 4 μM lycopene	No	Murine prostate adenocarcinoma cells TRAMP-C2 [49] treated in vitro with a novel antioxidant FruHis from tomato [50].
**Third**	Drosophila melanogaster	CF (Control Female), CM (Control Male), HMF (H83M2 Female), and HMM (H83M2 Male)	Three biological replicates	H83M2 is a transgenic stock number, which ectopically expresses MSL2 protein in females [14].
**Fourth**	Drosophila melanogaster	CF (Control Female), CM (Control Male), and mF (Meta Female)	Three biological replicates	Meta female is the triple X female [15].
**Fifth**	Arabidopsis thaliana	Columbia wild-type and *hae*-3 *hsl*2-3 mutants	Three biological replicates	There were two fastq files for each sample. One was solexa 1.3 quals output, and the other was Casava 1.8 output. The result files Col and *hae*-3 *hsl*2-3 were generated from solexa 1.3 quals outputs and the result files Col_qtrim and *hae*-3 *hsl*2-3_qtrim were generated from Casava 1.8 outputs [51].
**Sixth**	Human	1Sfesrrb, 2pc3, 3DY131, and 4ctrl	Two biological replicates	Samples 1Sfesrrb and 2pc3 were from Experiment 1, and samples 3DY131 and 4ctrl were from Experiment 2. 2pc3 was control for Experiment 1, and 1Sfesrrb was forced to express human Estrogen Related Receptor beta (esrrb), confirmed by real-time PCR. 4ctrl was control for experiment 2. In 3DY131, Esrrb agonist DY131 was added to the culture of DU145 cells at concentration of 3uM for 12 hours.

### 1. Results of mapping RNA-Seq reads to a reference genome

RNAMiner used TopHat2 [8] and Bowtie [44] to map RNA-Seq reads in the first and second data sets to the *Mouse* reference genome (mm9) in the UCSC genome browser [26] in conjunction with the RefSeq genome reference annotation (mm9) [27], map RNA-Seq reads in the third and fourth data sets to the *Drosophila melanogaster* reference genome (dm3) in the UCSC genome browser [26] in conjunction with the RefSeq genome reference annotation (dm3) [27], map RNA-Seq reads in the fifth data set to the *Arabidopsis thaliana* reference genome (ftp://ftp.arabidopsis.org/home/tair/Sequences/whole_ chromosomes/) in conjunction with the *Arabidopsis thaliana* genome reference annotation (ftp://ftp.arabidopsis.org/home/tair/Genes/TAIR10_genome_release/TAIR10_gff3/), and used TopHat2 [8] and Bowtie2 [9] to map RNA-Seq reads in the sixth data set to the *Homo sapiens* reference genomes (hg19) in the UCSC genome browser [26] in conjunction with the RefSeq genome reference annotation (hg19) [27]. Tables 4–9 show the mapping statistics of six sets of RNA-Seq data. Overall, more than 70% of reads were mapped to the genome successfully. Particularly, a very high mapping rate (~97%) was reached on the sixth data set. These mapping success rates were within the reasonable range, suggesting the good quality of the data and the correctness of the mapping process. This reads mapping process reduced the size of data by several orders of magnitude.

**Table 4:** Mapping statistics of the first set of RNA-Seq data of mouse.

Samples	# reads	# reads mapped to unique sites	# reads mapped to multiple sites	# reads failed to map	# filtered reads	Percentage of reads mapped to genome
**Mutant, Control**	22,053,527	14,120,075	2,577,992	5,330,066	25,394	75.72%
**Wild-type, Control**	29,483,443	19,560,525	3,077,978	6,809,626	35,314	76.78%
**Mutant, CDDO**	16,050,830	10,500,068	1,832,101	3,699,982	18,679	76.83%
**Wild-type, CDDO**	26,643,277	17,185,336	2,840,999	6,585,400	31,542	75.16%
**Mutant, Sutherlandia**	37,321,607	23,776,732	3,690,121	9,813,200	41,554	73.60%
**Wild-type, Sutherlandia**	27,678,509	18,150,349	2,717,683	6,777,541	32,936	75.39%
**Mutant, Elderberry**	25,750,508	17,155,488	2,631,750	5,932,836	30,434	76.84%
**Wild-type, Elderberry**	24,036,293	15,882,208	2,386,226	5,738,437	29,422	76.00%

**Table 5:** Mapping statistics of the second set of RNA-Seq data of mouse.

Samples	# reads	# reads mapped to unique sites	# reads mapped to multiple sites	# reads failed to map	# filtered reads	Percentage of reads mapped to genome
**2A**	12,390,167	9,016,108	1,513,122	1,849,425	11,512	84.98%
**2B**	11,760,788	8,220,731	1,445,292	2,083,834	10,931	82.19%
**2C**	9,481,395	6,892,027	1,178,753	1,402,253	8,362	85.12%
**2D**	19,450,682	13,849,985	2,406,684	3,176,235	17,778	83.58%
**2E**	11,743,452	8,381,763	1,418,645	1,932,480	10,564	83.45%
**2F**	12,104,053	8,692,100	1,510,391	1,890,846	10,716	84.29%
**2G**	13,301,646	9,427,606	1,642,257	2,219,819	11,964	83.22%
**2H**	15,766,959	11,158,652	1,950,974	2,643,055	14,278	83.15%
**3A**	22,688,673	16,126,579	2,773,025	3,768,846	20,223	83.30%
**3B**	20,352,253	14,506,676	2,503,008	3,324,616	17,953	83.58%
**3C**	20,301,445	14,486,410	2,401,849	3,394,815	18,371	83.19%
**3D**	14,985,494	10,729,926	1,876,610	2,365,106	13,852	84.12%

**Table 6:** Mapping statistics of the third set of RNA-Seq data of Drosophila melanogaster.

Samples	# reads	# reads mapped to unique sites	# reads mapped to multiple sites	# reads failed to map	# filtered reads	Percentage of reads mapped to genome
**CF1**	51,144,998	45,977,130	900,427	4,241,077	26,364	91.66%
**CF2**	81,302,211	74,246,307	1,415,608	5,590,921	49,375	93.06%
**CF3**	123,512,038	108,573,161	1,797,884	13,075,375	65,618	89.36%
**CM1**	77,424,855	70,221,478	1,520,820	5,642,533	40,024	92.66%
**CM2**	61,946,818	55,327,486	1,103,757	5,477,117	38,458	91.10%
**CM3**	69,294,584	61,985,415	1,518,519	5,750,241	40,409	91.64%
**HMF1**	85,587,833	78,370,743	1,323,717	5,849,024	44,349	93.11%
**HMF2**	44,339,865	39,020,383	701,810	4,593,648	24,024	89.59%
**HMF3**	75,974,183	68,654,562	1,302,500	5,973,758	43,363	92.08%
**HMM1**	74,429,022	67,318,010	1,682,459	5,382,557	45,996	92.71%
**HMM2**	68,985,281	61,450,714	1,302,852	6,195,229	36,486	90.97%
**HMM3**	76,796,015	69,056,721	1,578,337	6,116,669	44,288	91.98%

**Table 7:** Mapping statistics of the fourth set of RNA-Seq data of Drosophila melanogaster.

Samples	# reads	# reads mapped to unique sites	# reads mapped to multiple sites	# reads failed to map	# filtered reads	Percentage of reads mapped to genome
**CF1**	83,480,611	56,708,379	2,163,167	24,557,803	51,262	70.52%
**CF2**	56,660,705	42,714,627	1,398,576	12,513,930	33,572	77.86%
**CF3**	67,314,472	50,492,765	1,681,644	15,100,773	39,290	77.51%
**CM1**	50,000,247	38,470,206	1,206,401	10,291,223	32,417	79.35%
**CM2**	70,869,571	53,559,942	1,657,406	15,608,111	44,112	77.91%
**CM3**	68,530,284	51,799,947	1,627,155	15,061,138	42,044	77.96%
**mF1**	78,004,841	61,015,420	2,721,937	14,235,541	31,943	81.71%
**mF2**	51,629,214	40,273,082	1,573,248	9,745,829	37,055	81.05%
**mF3**	75,882,842	59,657,154	2,740,131	13,454,327	31,230	82.23%

**Table 8:** Mapping statistics of the fifth set of RNA-Seq data of Arabidopsis thaliana.

Samples	# reads	# reads mapped to unique sites	# reads mapped to multiple sites	# reads failed to map	# filtered reads	Percentage of reads mapped to genome
**Col_1**	27,725,818	24,853,210	973,400	1,897,394	1,814	93.15%
**Col_2**	34,323,205	30,712,426	1,319,275	2,289,425	2,079	93.32%
**Col_3**	27,486,337	24,759,189	836,816	1,888,591	1,741	93.12%
**Col_1_qtrim**	17,555,221	15,965,329	636,099	953,687	106	94.57%
**Col_2_qtrim**	22,064,711	20,041,732	876,453	1,146,446	80	94.80%
**Col_3_qtrim**	17,459,673	15,956,994	548,720	953,897	62	94.54%
***hae*-3 *hsl*2-3_1**	26,356,053	23,676,670	886,816	1,790,930	1,637	93.20%
***hae*-3 *hsl*2-3_2**	20,998,406	18,793,308	727,901	1,475,840	1,357	92.97%
***hae*-3 *hsl*2-3_3**	28,372,647	25,669,013	914,982	1,786,946	1,706	93.70%
***hae*-3 *hsl*2-3_1_qtrim**	16,641,162	15,168,566	578,226	894,325	45	94.63%
***hae*-3 *hsl*2-3_2_qtrim**	13,167,066	11,963,242	473,323	730,435	66	94.45%
***hae*-3 *hsl*2-3_3_qtrim**	17,927,078	16,467,776	597,035	862,219	48	95.19%

**Table 9:** Mapping statistics of the sixth set of RNA-Seq data of human.

Samples	# reads	# reads mapped to unique site	# reads mapped to multiple sites	# reads failed to map	# filtered reads	Percentage of reads mapped to genome
**1Sfesrrb-2**	17,448,758	16,392,693	692,905	363,022	138	97.92%
**1Sfesrrb-3**	16,228,533	15,239,649	662,064	326,704	116	97.99%
**2pc3-1**	15,582,276	14,641,199	626,397	314,544	136	97.98%
**2pc3-3**	17,066,953	16,009,327	707,445	350,042	139	97.95%
**3DY131-1**	17,130,579	15,966,495	806,969	357,004	111	97.92%
**3DY131-2**	15,500,204	14,623,868	576,060	300,168	108	98.06%
**4ctrl-1**	19,117,412	17,885,236	858,147	373,922	107	98.04%
**4ctrl-2**	16,269,465	15,280,726	663,813	324,810	116	98.00%

### 2. Gene expression values calculated from the reads mapping data

**Fig 6:**
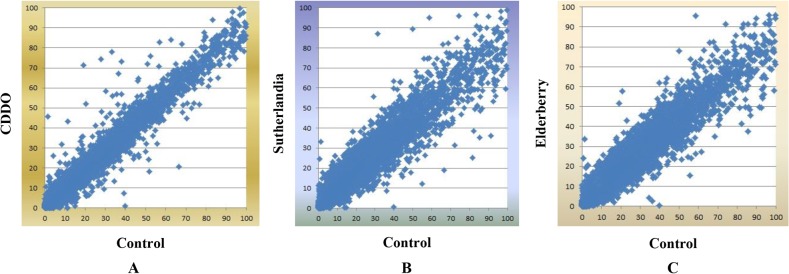
Three gene expression plots in the first data set. These plots are for comparisons between Control and each treatment in mutant mouse. The x-axis represents Control and the y-axis represents CDDO treatment in A, Sutherlandia treatment in B, and Elderberry treatment in C. We used gene expression values calculated by MULTICOM-MAP to make the plots. The range of these values was constrained to [0, 100] while keeping the original ratios in order to make these figures readable.

**Fig 7:**
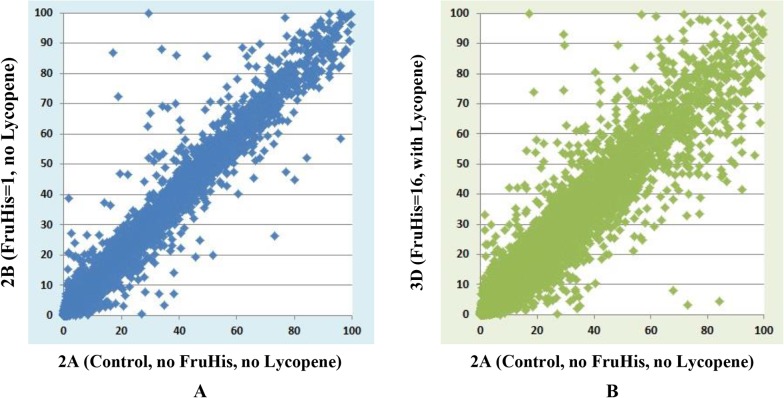
Two gene expression plots in the second data set. These plots are for comparisons between 2A and 2B, between 2A and 3D. The x-axis represents 2A (Control, no FruHis, no Lycopene) and the y-axis represents 2B (FruHis = 1, no Lycopene) in A and 3D (FruHis = 16, with Lycopene) in B. We used gene expression values calculated by MULTICOM-MAP to make the plots. The range of these values was constrained to [0, 100] while keeping the original ratios in order to make these figures readable.

RNAMiner removed reads that mapped to multiple locations on a reference genome from the mapping data. The gene expression values were calculated by Cufflinks [10], MULTICOM-MAP [14,15,16], and HTSeq [11] on the remaining RNA-Seq reads mapped to unique locations on the genome. Compiling reads mappings into gene expression values generates an overall profile of the expression levels of most genes in a transcriptome, which can reduce the size of dataset by about one thousand-fold (i.e., from gigabytes to megabytes) in our experiments. The compilation process transforms the raw data into meaningful expression profiles of genes. For example, three gene expression plots for comparisons between Control and each treatment in mutant mouse in the first data set are shown in Fig 6, and two gene expression plots for comparisons between 2A and 2B, between 2A and 3D in the second dataset are shown in Fig 7. In these figures, gene expression values calculated by MULTICOM-MAP were used, and the range of these values was constrained to [0, 100] while keeping the original ratios in order to make these figures readable. Usually, the points beyond the diagonal are candidates of differentially expressed genes.

**Fig 8:**
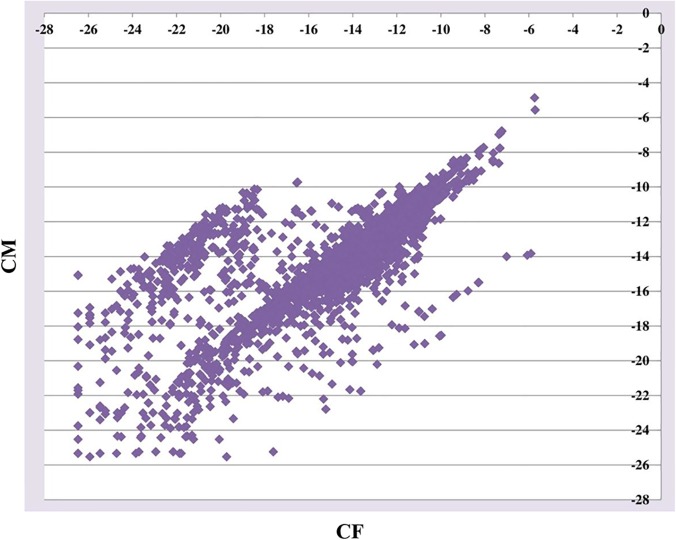
One gene expression plot in the third data set. The plot is for the comparison between CF and CM. The x-axis represents CF and the y-axis represents CM. We used gene expression values calculated by MULTICOM-MAP to make the plot. The raw counts were transformed by log_2_ in order to make the figure readable.

**Fig 9:**
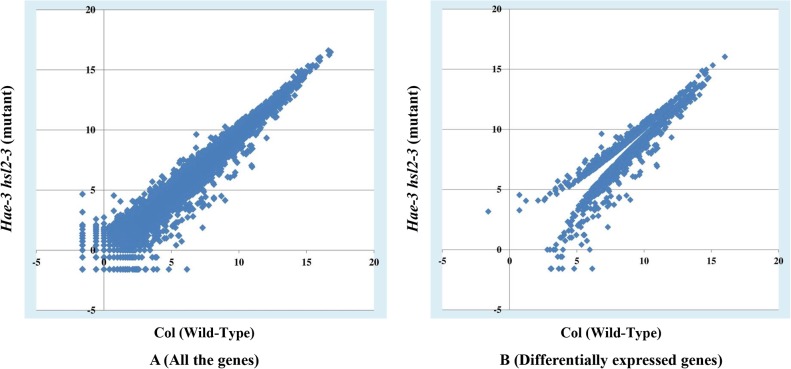
Two gene expression plots in the fifth data set. These plots are for the comparison between Col and *hae*-3 *hsl*2-3. The x-axis represents Col (Wild-Type) and the y-axis represents *hae*-3 *hsl*2-3 (mutant). The left plot visualizes the expression values of all the genes, and the right one displays the expression values of differentially expressed genes. The gene expression values were calculated by MULTICOM-MAP. The raw counts were transformed by log_2_.

**Fig 10:**
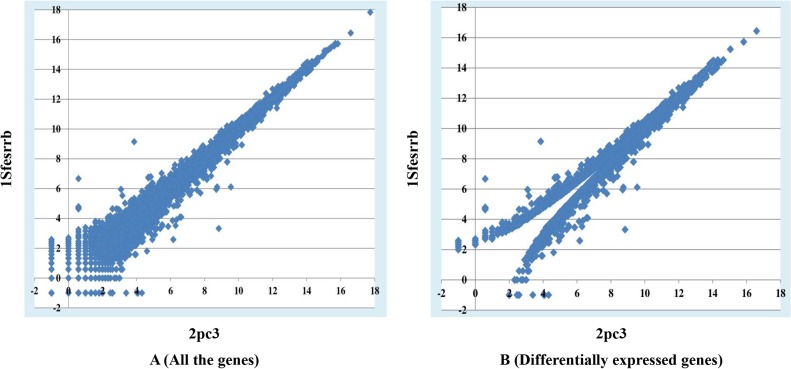
Two gene expression plots in the sixth data set. These plots are for the comparison between 2pc3 and 1Sfesrrb. The x-axis represents 2pc3 and the y-axis represents 1Sfesrrb. The left plot visualizes the expression values of all the genes, and the right one displays the expression values of differentially expressed genes. The gene expression values were calculated by MULTICOM-MAP. The raw counts were transformed by log_2_.

MULTICOM-MAP and HTSeq were used to calculate the raw read counts in the third and fourth sets of data. The counts were normalized by dividing them by the total number of uniquely mapped reads in the replicate. The normalized count of a gene was an indicator of the relative expression level of the gene in the replicate. The normalized counts of a gene in multiple replicates of a sample were further averaged and used as the measure of the relative expression level of the gene in the sample. Fig 8 shows one gene expression plot for the comparison between CF (Control Female) and CM (Control Male) in the third data set. In this figure, gene expression values were calculated by MULTICOM-MAP, and the values were transformed by log_2_ in order to make the figure readable. Two gene expression plots for the comparison between Col (Wild-Type) and *hae*-3 *hsl*2-3 (mutant) in the fifth data set are illustrated in Fig 9, and two gene expression plots for the comparison between 2pc3 and 1Sfesrrb in the sixth data set are illustrated in Fig 10. The left plot in each figure was generated from all the genes, and the right one was generated from differentially expressed genes. The gene expression values were calculated by MULTICOM-MAP and normalized by log_2_. According to the two plots in Figs 9 and 10, the distribution of expression values of differentially expressed genes is quite different than that of the rest of the genes.

### 3. Differentially expressed genes identified from the RNA-Seq data

**Fig 11:**
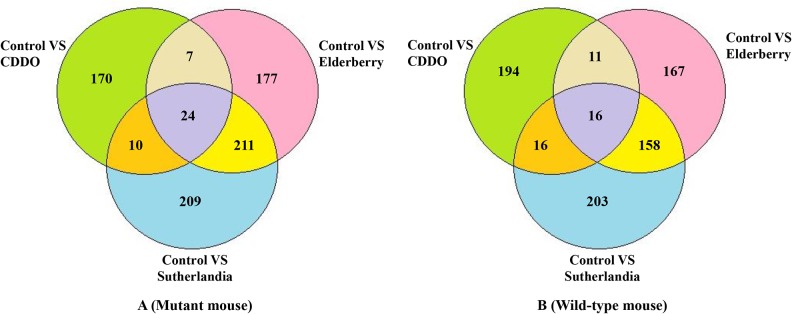
The number of differentially expressed genes in the first data set. These numbers were calculated for different pairs of comparisons between Control and each treatment, and their overlaps in both mutant mouse and wild-type mouse cells. The differentially expressed genes in each comparison were derived from the overlaps of three sets of differentially expressed genes generated by Cuffdiff, MULTICOM-MAP+edgeR, and MULTICOM-MAP+DESeq.

**Fig 12:**
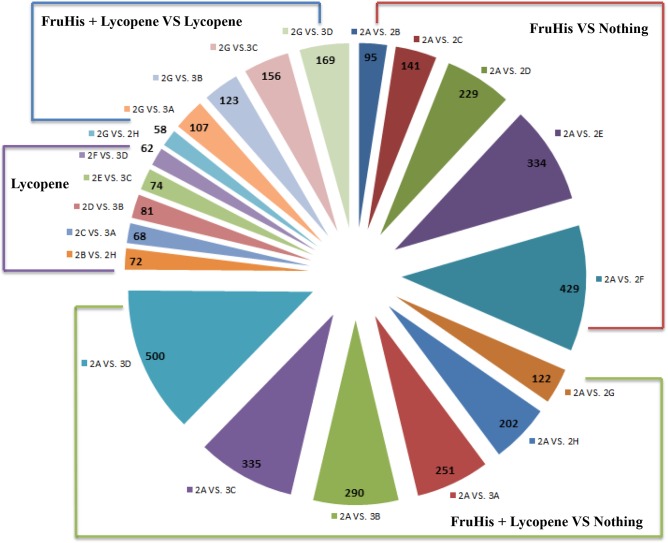
The number of differentially expressed genes for each pairwise comparison in the second data set. The differentially expressed genes in each comparison were derived from the overlaps of three sets of differentially expressed genes generated by Cuffdiff, MULTICOM-MAP+edgeR, and MULTICOM-MAP+DESeq.

RNAMiner identified differentially expressed genes between control and each treatment using Cuffdiff [10], edgeR [12], and DESeq [13]. The threshold of p-value was set to 0.05 to select differentially expressed genes. For example, the number of differentially expressed genes for each comparison and their overlaps in both mutant mouse and wild-type mouse in the first data set are shown in Fig 11. The number of differentially expressed genes for each comparison in the second data set is shown in Fig 12. These differentially expressed genes were derived from the overlaps of three sets of differentially expressed genes separately identified by Cuffdiff, MULTICOM-MAP+edgeR, and MULTICOM-MAP+DESeq. As shown in Fig 12, the number of differentially expressed genes increased with the increase of FruHis concentration in the absence or presence of 4 μM lycopene.

**Fig 13:**
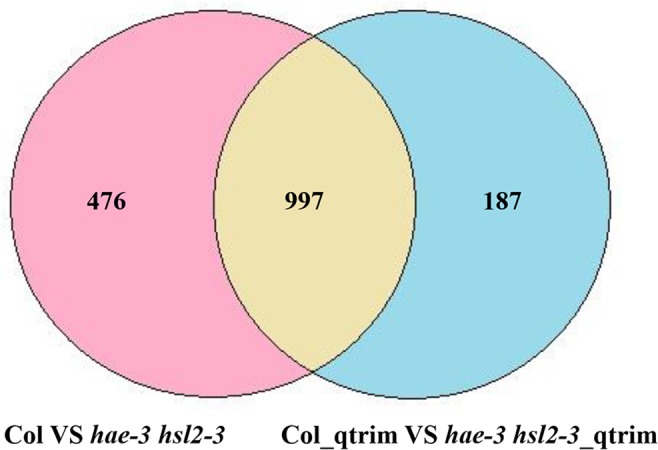
The number of differentially expressed genes in the fifth data set. These numbers were calculated for two comparisons between Col and *hae*-3 *hsl*2-3, between Col_qtrim and *hae*-3 *hsl*2-3_qtrim, and their overlap. The differentially expressed genes in each comparison were derived from the overlaps of three sets of differentially expressed genes generated by Cuffdiff, MULTICOM-MAP+edgeR, MULTICOM-MAP+DESeq.

The number of differentially expressed genes for two comparisons between Col (Wild-Type) and *hae*-3 *hsl*2-3 (mutant), between Col_qtrim (Wild-Type) and *hae*-3 *hsl*2-3_qtrim (mutant), and their overlaps in the fifth data set are shown in Fig 13. These differentially expressed genes were derived from the overlaps of three sets of differentially expressed genes generated separately by Cuffdiff, MULTICOM-MAP+edgeR, MULTICOM-MAP+DESeq. We also identified differentially expressed genes for two comparisons: between 2pc3 and 1Sfesrrb, between 4ctrl and 3DY131 in the sixth data set using edgeR based on read counts calculated by MULTICOM-MAP. EdgeR identified 6,210 differentially expressed genes for the comparison between 2pc3 and 1Sfesrrb, and 590 differentially expressed genes for the comparison between 4ctrl and 3DY131. On the RNAMiner web service, users can select different p-value (or q-value) thresholds to select a specific number of differentially expressed genes according to their needs. In addition to generating the testable biological hypotheses (e.g. gene targets for experimental testing), differential gene expression analysis generally reduces the size of data by about two folds, shifting point of interest from almost all the genes in a genome to a small portion of genes most relevant to the biological experiment.

### 4. Predicted functions of differentially expressed genes

**Fig 14:**
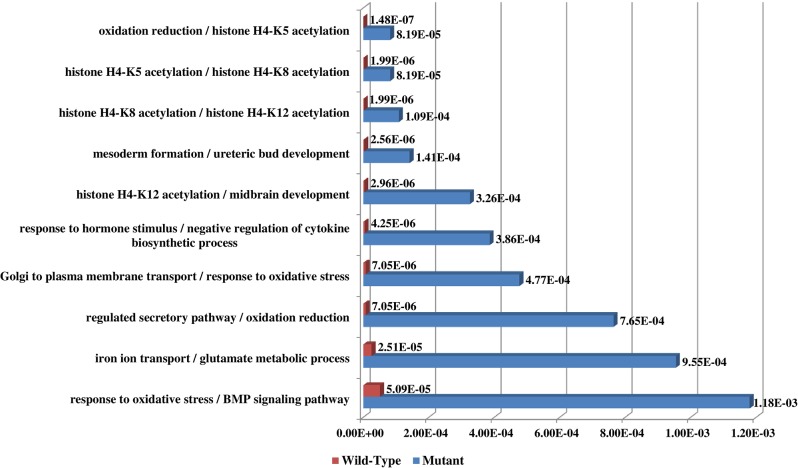
Top 10 biological process functions in the first data set. These functions were predicted for the comparison between Control and CDDO in both mutant mouse and wild-type mouse cells. Red bars denote the p-values of the top 10 predicted functions for wild-type mouse and blue bars denote the p-values of the top 10 predicted functions for mutant mouse. The number besides each bar is the significance of enrichment (p-value) of the predicted function. The p-value was calculated by Fisher’s exact test. The function names of wild-type mouse and mutant mouse are listed on the left separated by “/”. The two comparisons have five common biological processes among the top 10 biological processes.

**Fig 15:**
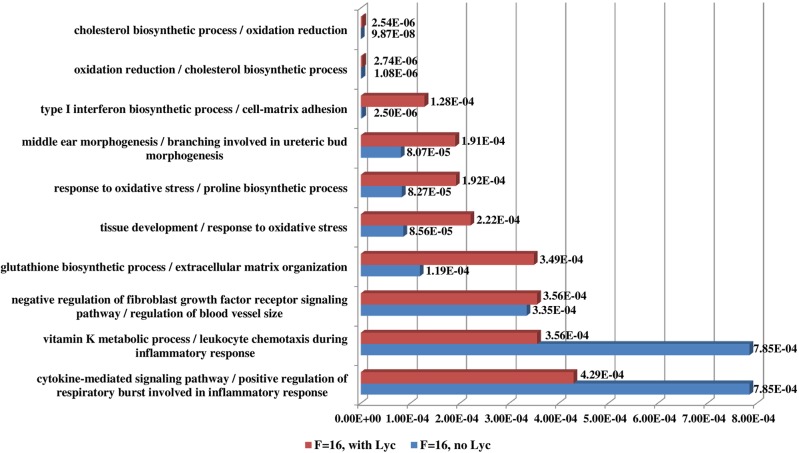
Top 10 biological process functions in the second data set. These functions were predicted for two comparisons between 2A (Control, no FruHis, no Lycopene) and 2F (FruHis = 16 without Lycopene), between 2A and 3D (FruHis = 16 with Lycopene). Red bars denote the p-values of the top 10 predicted functions for the comparison between 2A and 3D and blue bars denote the p-values of the top 10 predicted functions for the comparison between 2A and 2F. The number besides each bar is the significance of enrichment (p-value) of the predicted function. The p-value was calculated by Fisher’s exact test. The function names of the two comparisons between 2A and 3D, between 2A and 2F are listed on the left separated by “/”. The two comparisons have three common biological processes among the top 10 biological processes.

**Fig 16:**
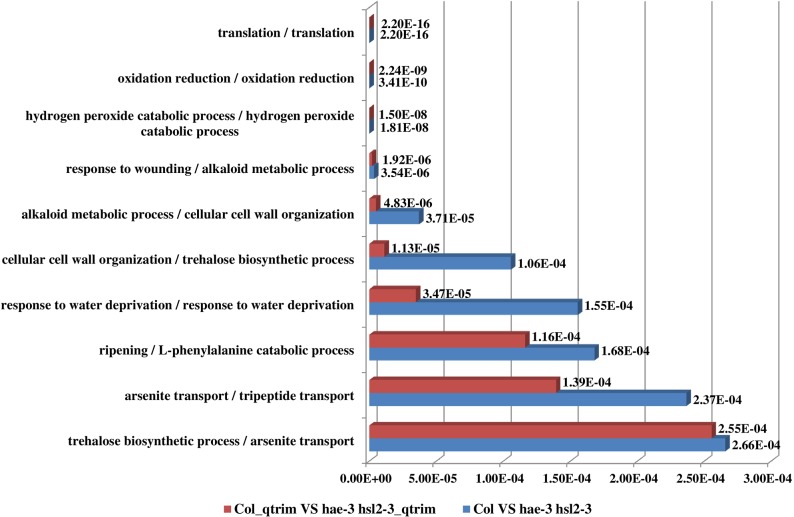
Top 10 biological process functions in the fifth data set. These functions were predicted for two comparisons between Col and *hae*-3 *hsl*2-3, between Col_qtrim and *hae*-3 *hsl*2-3_qtrim. Red bars denote the p-values of the top 10 predicted functions for the comparison between Col_qtrim and *hae*-3 *hsl*2-3_qtrim and blue bars denote the p-values of the top 10 predicted functions for the comparison between Col and *hae*-3 *hsl*2-3. The number besides each bar is the significance of enrichment (p-value) of the predicted function. The p-value was calculated by Fisher’s exact test. The function names of the two comparisons between Col_qtrim and *hae*-3 *hsl*2-3_qtrim, between Col and *hae*-3 *hsl*2-3 are listed on the left separated by “/”. The two comparisons have 8 common biological processes among the top 10 biological processes.

RNAMiner predicted functions of differentially expressed genes using MULTICOM-PDCN. The predicted function terms were ranked by their significance of enrichment among the differentially expressed genes. For example, Fig 14 shows the top 10 most significantly enriched biological process functions for the comparison between Control and CDDO in both mutant mouse and wild-type mouse in the first data set. The two comparisons have 5 common biological processes in the top 10 biological processes. The top 10 biological process functions for two comparisons between 2A and 2F (FruHis = 16 without Lycopene), between 2A and 3D (FruHis = 16 with Lycopene) in the second data set are shown in Fig 15. The two comparisons have 3 common biological processes in the top 10 biological processes. The top 10 biological process functions for the comparison between Col (Wild-Type) and *hae*-3 *hsl*2-3 (mutant) in the fifth data set are reported in Fig 16, and the two comparisons share 8 common biological processes in the top 10 biological processes. In these figures, the number besides each column is p-value of the enrichment of each predicted function.

Although the step of gene function analysis does not substantially reduce the size of data physically, it can logically summarize hundreds of differentially expressed genes into a small number (i.e., tens) of biological processes activated or deactivated in the biological experiment which sheds light into the potential biological mechanism relevant to the experiment.

### 5. Constructed gene regulatory networks

**Fig 17:**
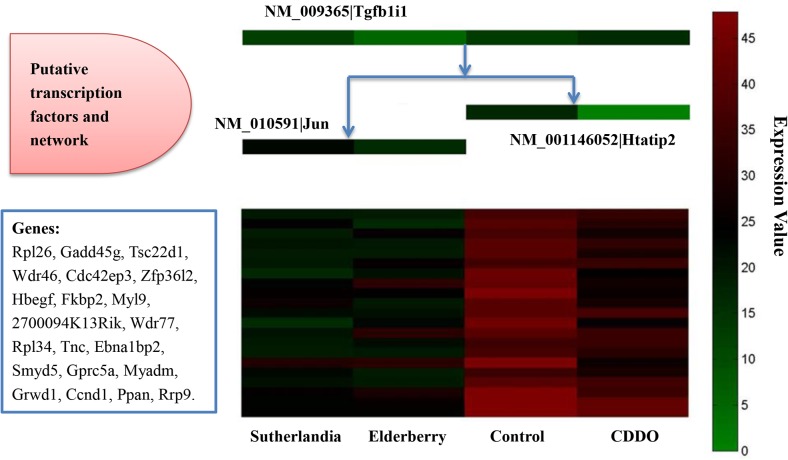
One repression gene regulatory module in mutant mouse cells in the first data set. The expression correlation score of the module was 0.85. The decision tree on the middle top illustrates how three putative transcription factors (Tgfb1i1, Htatip2, Jun) may collaboratively regulate the cluster of co-expressed genes in the middle bottom, where each row denotes a gene listed in the bottom left box and each column denotes one of four biological conditions (i.e. Control, CDDO, Sutherlandia, and Elderberry). The levels of gene expression values were represented by different colors ranging from lowest (green) to highest (red). The expression of the genes in the cluster under each condition is predicted to be regulated according to the expression levels of transcription factors listed on top of the condition. For example, under Sutherlandia treatment, the relatively low expression of Tgfbli1 and the medium expression of Jun caused the repression of the group of genes.

**Fig 18:**
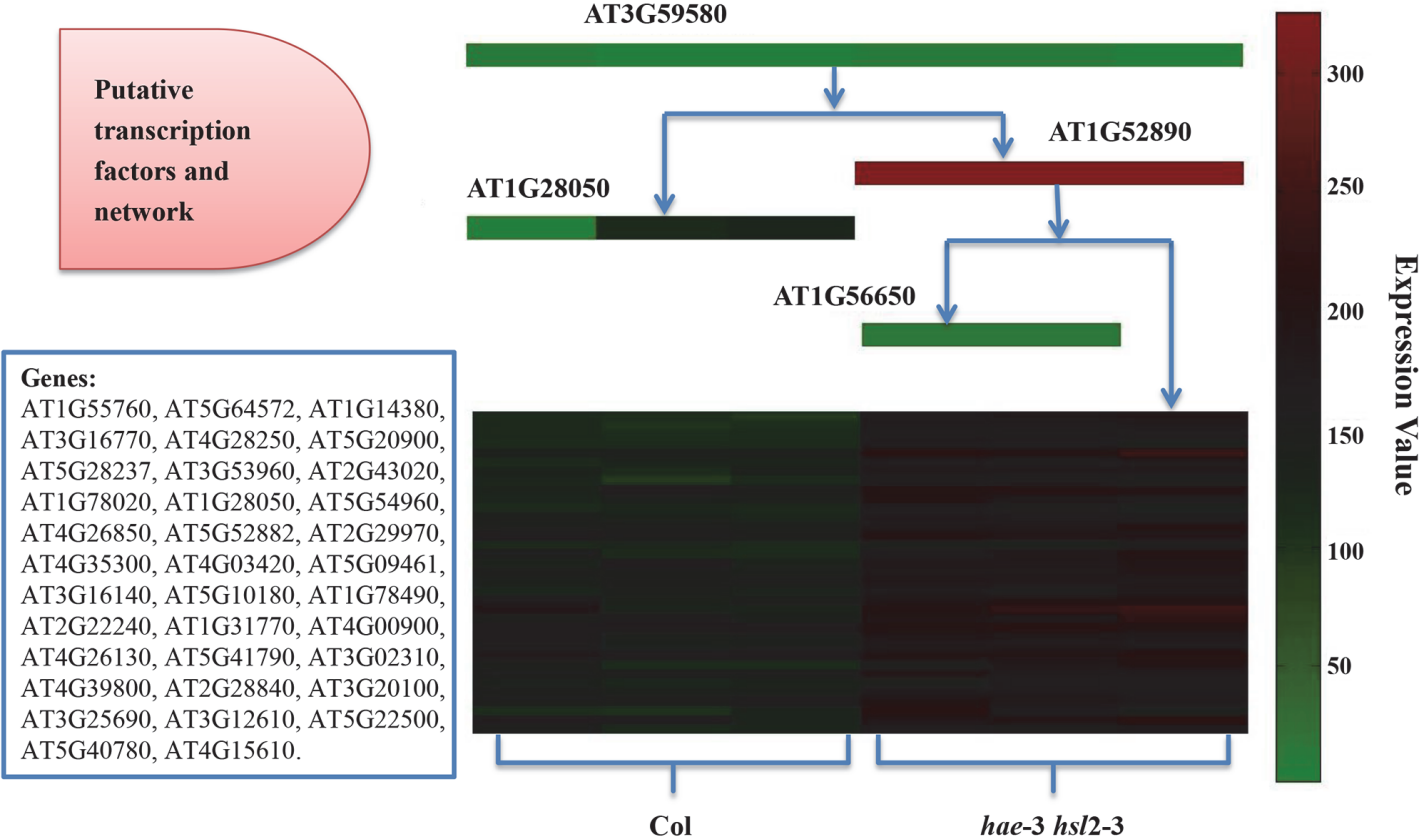
One activation gene regulatory module in the fifth data set. The gene regulatory module was constructed for the comparison between Col (Wild-Type) and *hae*-3 *hsl*2-3 (mutant), and the expression correlation score of the module was 0.85. The decision tree on the middle top illustrates how four putative transcription factors (AT3G59580, AT1G28050, AT1G52890, AT1G56650) may collaboratively regulate the cluster of co-expressed genes in the middle bottom, where each row denotes a gene listed in the bottom left box and each column denotes one of six biological replicates of two samples (i.e. Col and *hae-3 hsl2-3*). The levels of gene expression values were represented by different colors ranging from lowest (green) to highest (red). The expression of the genes in the cluster under each sample is predicted to be regulated according to the expression levels of transcription factors listed on top of the condition. For example, under the first replicate of Col, the low expression of AT3G59580 and the low expression of AT1G28050 caused the repression of the group of genes.

RNAMiner used MULTICOM-GNET [19,20] to construct gene regulatory networks based on differentially expressed genes and transcription factors. For example, a repression gene regulatory module with expression correlation 0.85 in mutant mouse in the first data set is illustrated in Fig 17. This module was comprised of 21 differentially expressed genes. Three transcription factors: Tgfb1i1, Htatip2, and Jun, were predicted to collaboratively regulate this group of genes. An activation gene regulatory module for the comparison between Col (Wild-Type) and *hae*-3 *hsl*2-3 (mutant) with expression correlation 0.85 in the fifth data set is shown in Fig 18. This module was comprised of 35 differentially expressed genes. Four transcription factors, AT3G59580, AT1G56650, AT1G28050, and AT1G52890, were predicted to collaboratively regulate this group of genes.

**Fig 19:**
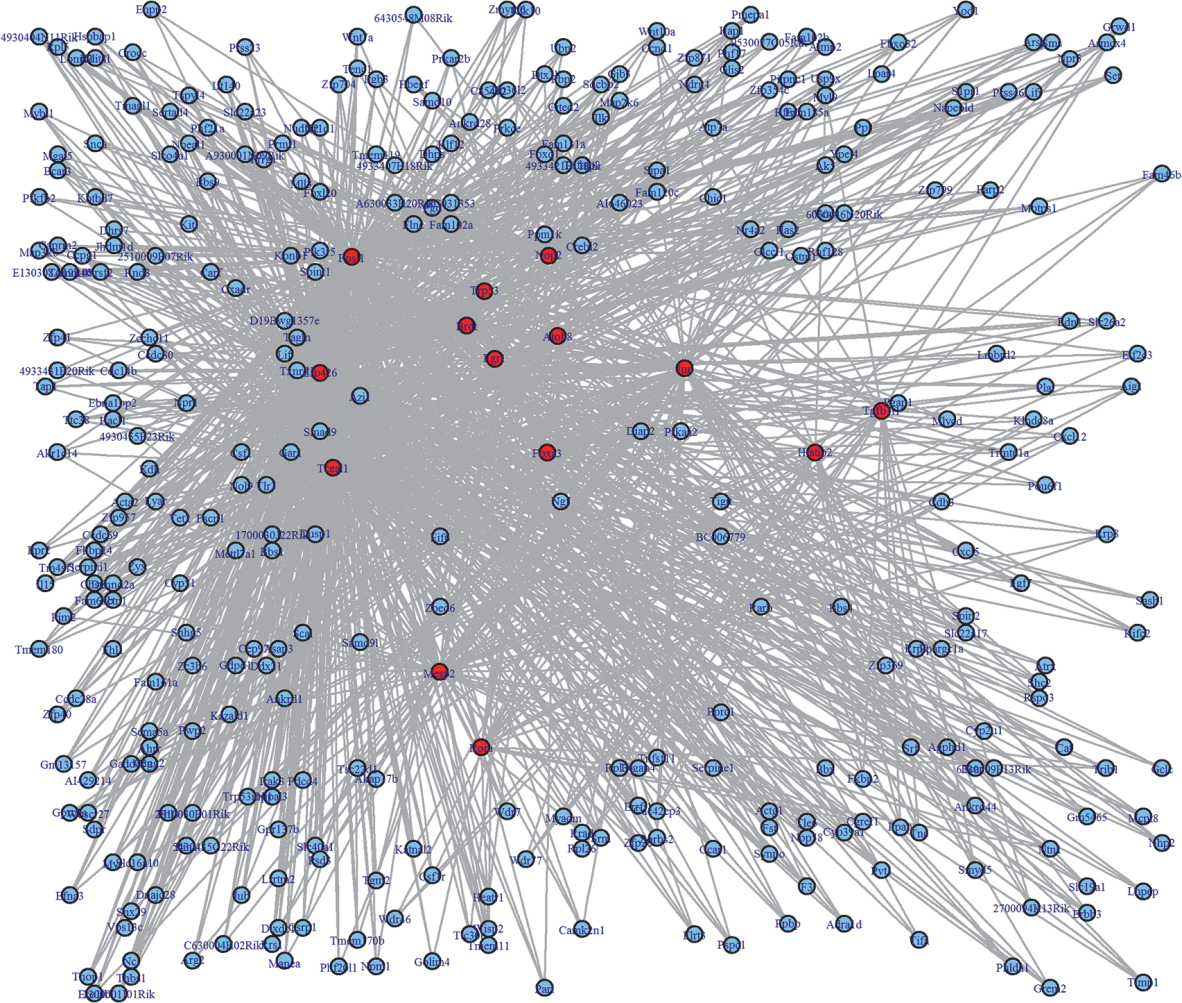
A visualized global gene regulatory network on the first dataset. The network includes all the gene regulatory relationships between and within top 10 gene regulatory modules ranked by expression correlation scores on the first dataset. Blue nodes represent target genes, and red nodes represent transcription factors which regulate the target genes. Each edge represents a regulatory relationship between a transcription factor and a gene.

RNAMiner also used a R package “igraph” [43] to visualize gene regulatory networks by linking the regulatory relationships between and within all the gene regulatory modules predicted by MULTICOM-GNET together. Fig 19 shows a gene regulatory network representing the regulatory relationships of top 10 gene regulatory modules ranked by expression correlation scores on the first data set. There are 14 transcription factors (red nodes), 338 genes (blue nodes), and 1,280 edges (regulatory relationships) in the network.

The step of gene regulatory network reconstruction condenses hundreds of differentially expressed genes and their expression data into dozens of valuable gene regulatory modules, which may reveal the underlying biological mechanism controlling the expression in the biological experiment. The network modules not only provide the human comprehensible interpretation of the gene expression levels, but also the important transcription factors and their target genes that are very valuable for generating hypotheses for new biological experiments.

## Use of the RNAMiner Web Service

**Fig 20:**
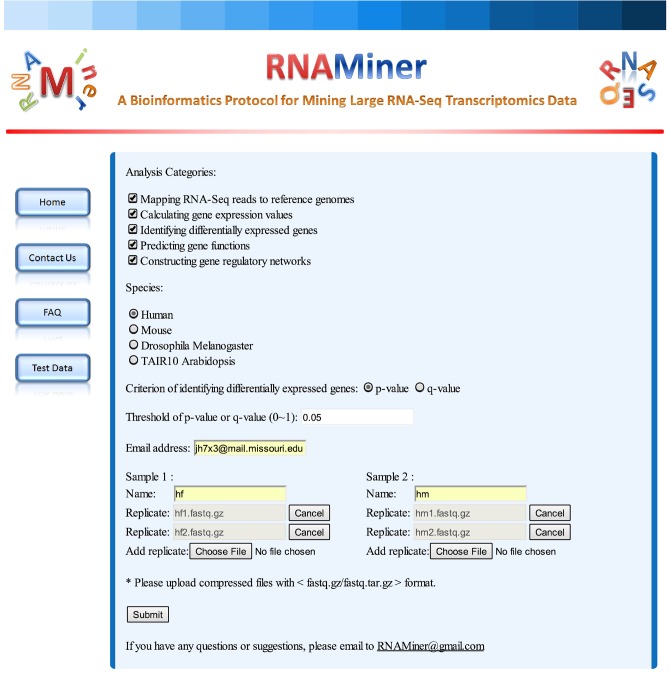
The home page of the RNAMiner web service. Users can submit requests on the home page of the RNAMiner web service, and also can learn how to use RNAMiner, find contact information, and download the test data by clicking the navigation buttons on left.

The RNAMiner web service (Fig 20) is available at http://calla.rnet.missouri.edu/rnaminer/index.html. Users can submit requests on the home page and receive an email with a link to the data analysis results.

### 1. Submit a request

1. Prepare RNA-Seq reads files (.fastq). The acceptable formats by the RNAMiner web service include “.fastq.gz” and “.fastq.tar.gz”.
2. Choose the analysis categories. Each category needs the results in the previous categories. If one category is chosen, the previous categories will be executed automatically. For example, if “predicting gene functions” is chosen, the first three categories will be executed automatically.
3. Choose the species. RNAMiner can analyze RNA-Seq data on four species: *Human*, *Mouse*, *Drosophila melanogaster*, and *Arabidopsis thaliana*.
4. Choose criterion of identifying differentially expressed genes. It is p-value or q-value.
5. Set threshold of p-value or q-value for identifying differentially expressed genes. The value should be between 0 and 1. The default value is 0.05.
6. Input email address. An email with a link to the data analysis results will be sent to this email address when the data analysis is finished.
7. Input sample names.
8. Upload reads files. The last three categories request users to upload reads files for both two samples. Users can upload more than one reads files for each sample.
9. Click “Submit”.

**Fig 21:**
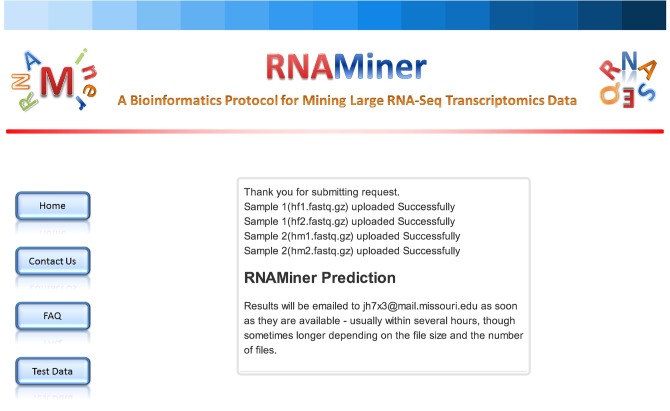
One web page showing the successful submission of one request. After one request is submitted successfully, one web page will be shown which informs users that the data is in process.

After a request is submitted successfully, one web page (Fig 21) will be shown saying the data is in process. If one user submitted one request to the RNAMiner web service and it is running or it is in the waiting queue, he/she cannot submit another request.

### 2. Receive the results

**Fig 22:**
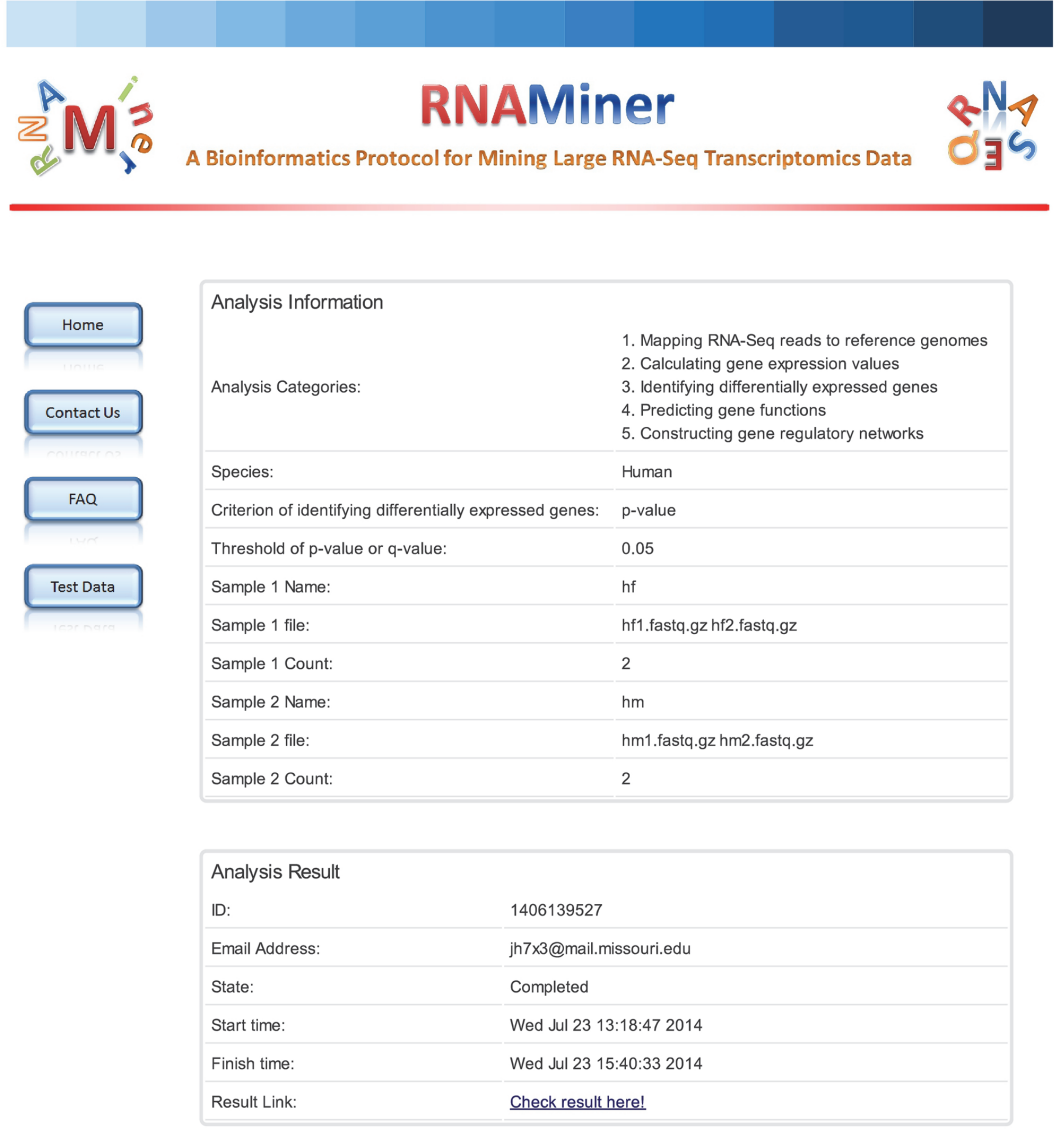
One web page with data analysis information and a result link. After the data analysis is finished, users will receive an email with a link to one web page with data analysis information and a result link. On this page, users can check the data analysis information and go to the result page.

**Fig 23:**
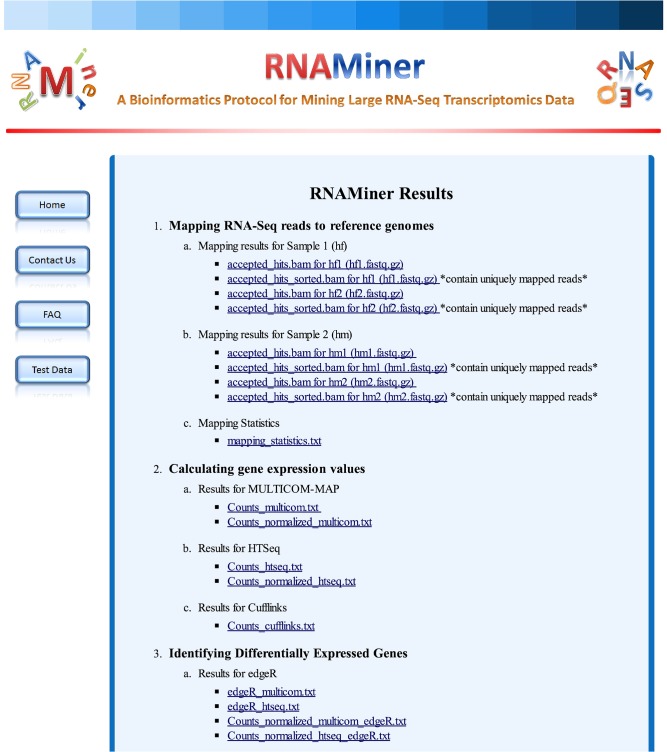
One web page with data analysis results. Users can view and download the data analysis results for each analysis category on this web page.

When the data analysis is finished, users will receive an email with a link to one web page (Fig 22) with the data analysis information and a result link. The result page (Fig 23) will be shown by clicking the result link. Users can view and download the analysis data on the result page.

The time expense of analyzing a set of RNA-Seq data by RNAMiner depends on how big the data is, how many reads files there are in the data set, and how many jobs there are in the waiting queue. Normally a data analysis can be finished by RNAMiner in several hours. However, the time expense will be longer if there are a lot of jobs in the waiting queue. Our server cannot handle too many jobs at the same time because of CPU and space limitations.

## Conclusions

The RNAMiner protocol and pipeline can progressively reduce the size of large datasets to produce valuable and comprehensible biological knowledge of manageable size, ranging from gene expression values, differentially expressed genes, gene function predictions, and gene regulatory networks. The test results on six RNA-Seq datasets of four different species help demonstrate its utility and versatility.

In order to further improve the quality of RNA-Seq data analysis, additional tools can be plugged into the RNAMiner protocol. In the future, we will add a high-speed RNA mapping tool—Gsnap [25] and a high-accuracy RNA mapping tool—Stampy [45] into the pipeline to map RNA reads to reference genomes. For identifying differentially expressed genes, we will include baySeq [46], ShrinkSeq [47], and NOISeq [48] into the pipeline in order to handle various sources of noise in RNA-Seq data even better. Furthermore, we will include an in-house tool of constructing biological networks from a group of co-expressed genes to reconstruct highly valuable metabolic networks and signal transduction networks for gene clusters identified by the RNAMiner protocol. Moreover, we will add the capability of analyzing the function of non-coding small RNAs into RNAMiner and use the information during the reconstruction of biological networks. The new improvements will be incorporated into the RNAMiner web service for the community to use.
